# Redox regulation of HIV-1: the thioredoxin pathway, oxidative metabolism, and latency control

**DOI:** 10.3389/fphys.2025.1651148

**Published:** 2025-09-09

**Authors:** Jesse F. Mangold, Talia H. Swartz

**Affiliations:** ^1^ Medical Scientist Training Program, Graduate School of Biomedical Sciences, Icahn School of Medicine at Mount Sinai, New York, NY, United States; ^2^ Division of Infectious Diseases, Department of Medicine, Icahn School of Medicine at Mount Sinai, New York, NY, United States

**Keywords:** HIV-1 latency, thioredoxin pathway, oxidative metabolism, Tat protein, redox homeostasis

## Abstract

Redox homeostasis is a critical determinant of HIV-1 pathogenesis, influencing viral entry, transcription, latency, and persistence in distinct cellular reservoirs. The thioredoxin (Trx) system, a central antioxidant pathway, modulates the redox state of transcription factors and viral proteins while buffering oxidative stress. Paradoxically, while oxidative signals can drive HIV-1 gene expression, the virus also co-opts host antioxidant systems, such as thioredoxin (Trx) and glutathione (GSH), to support its replication and survival. In this review, we examine the multifaceted roles of the Trx pathway in HIV-1 infection, highlighting how redox regulation influences transcriptional activation through NF-κB and AP-1, and modulates the function of viral proteins, such as Tat. We further explore how oxidative metabolism intersects with redox balance to influence latency, particularly through cell-type-specific mechanisms in CD4^+^ T cells and myeloid cells. Emerging insights into thioredoxin-interacting protein (TXNIP) reveal a critical interface between glucose metabolism, ROS signaling, and latency control. Notably, interventions targeting redox homeostasis—whether antioxidant or pro-oxidant—exert divergent effects depending on the cellular reservoir, underscoring the need for tailored therapeutic strategies. By integrating redox biology and immunometabolism, we outline potential avenues to either stabilize latency or induce viral reactivation in pursuit of an HIV-1 cure.

## Introduction

Redox homeostasis is intricately linked with the life cycle of Human Immunodeficiency Virus type 1 (HIV-1). Early observations revealed that altering the cellular redox balance could modulate HIV-1 replication: for example, addition of the antioxidant N-acetylcysteine (NAC) suppressed cytokine-driven viral replication *in vitro* (Roederer et al., 1990; Nakamura et al., 2002). This suggested that oxidative signals promote HIV-1 transcription, and sparked interest in redox-based therapeutic strategies (Roederer et al., 1992). Paradoxically, subsequent research has shown that the virus actively exploits the host’s antioxidant systems. The major cellular antioxidant pathways, including thioredoxin (Trx) and glutathione (GSH) systems, can promote HIV-1 infection while dampening effective immune responses (Benhar et al., 2016). Such findings underscore a complex role for redox biology in HIV-1 pathogenesis, in which reactive oxygen species (ROS) and reducing catalysts are involved in viral entry, the regulation of proviral transcription, and the maintenance of latency. Chronic HIV-1 infection, even in the setting of suppressive antiretroviral therapy, is associated with persistent oxidative stress and metabolic disturbances (Rodriguez et al., 2024). A deeper understanding of how the Trx pathway and oxidative metabolism intersect with HIV-1 transcriptional control and latency is critical, as it may reveal novel strategies to either “shock” the latent virus out of hiding or to “lock” it in deep latency. This review discusses the Trx redox system and oxidative metabolism in the context of HIV-1 pathogenesis, highlighting how redox regulation influences viral transcription and latency in different cell types.

## The thioredoxin pathway and redox homeostasis in HIV infection

Thioredoxin is a ubiquitous thiol-reducing protein that plays a central role in maintaining the intracellular redox balance. The Trx pathway comprises thioredoxin, thioredoxin reductase (TrxR), and peroxiredoxins, which use Trx to neutralize peroxides (Jin et al., 1997). TrxR is an NADPH-dependent selenoprotein that regenerates Trx to its reduced form. Together with GSH, the Trx system buffers cells against excessive ROS and regulates redox-sensitive signaling pathways. However, in HIV-1 infection, this pathway is dysregulated in a cell-specific manner. As early as 1992, Masutani et al. observed a loss of Trx-rich cells in the lymphoid tissues of patients with AIDS (Masutani et al., 1992), suggesting that cells with robust Trx expression might be depleted during disease progression. Conversely, in the monocyte-macrophage lineage, Trx levels can be maintained or even elevated in advanced HIV infection (Elbim et al., 2001). One study found that blood monocytes from asymptomatic people living with HIV (PLWH) had low Trx and high ROS production. In contrast, monocytes from late-stage patients showed restored Trx levels along with increased expression of anti-apoptotic Bcl-2 (Elbim et al., 1999; Aillet et al., 1998). Restoration of Trx and Bcl-2 in late disease could help explain why monocyte-macrophage counts remain relatively stable despite ongoing oxidative stress.

Thioredoxin is unique among antioxidants in that it also functions as a signaling molecule. Activated T cells can secrete Trx, and an oxidized truncated form of Trx (eosinophil cytotoxicity-enhancing factor, ECEF) has been found to exhibit cytokine-like activities (Newman et al., 1994). Intact Trx in the extracellular milieu tends to inhibit viral production, whereas the truncated form has been reported to enhance HIV replication (Newman et al., 1994). These findings imply that not only the presence of Trx, but also its redox state and form, are essential in modulating HIV-1 pathogenesis. In PLWH, intracellular GSH is often decreased, and important seleno-enzymes such as TrxR and glutathione peroxidase are downregulated (Gladyshev et al., 1999). HIV-1 Tat protein can directly contribute to this imbalance by suppressing the expression and activity of seleno-glutathione peroxidase, thereby impairing cellular ability to neutralize peroxides (Richard et al., 2001). HIV-1 infection perturbs the normal redox equilibrium such that infected cells experience higher ROS levels coupled with weakened antioxidant defenses (Perl and Banki, 2000).

**FIGURE 1:**
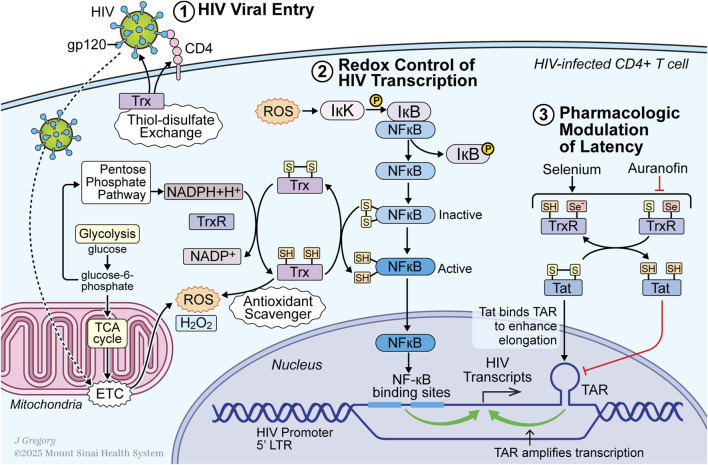
Redox regulation by the thioredoxin (Trx) pathway across key stages of HIV-1 infection. (1) Viral Entry: Cell-surface thioredoxin (Trx) catalyzes the reduction of disulfide bonds in the HIV-1 envelope glycoprotein gp120 and the host CD4 receptor, facilitating conformational changes required for membrane fusion. HIV-1 infection induces oxidative stress through disruption of the mitochondrial electron transport chain (ETC) and accumulation of reactive oxygen species (ROS). (2) Viral Transcription: Intracellular Trx translocates to the nucleus, where it reduces redox-sensitive cysteines in transcription factors such as nuclear factor kappa-light-chain-enhancer of activated B cells (NF-κB), enhancing HIV-1 gene expression. Trx also supports antioxidant defenses by sustaining ROS-scavenging systems. HIV Tat functions more effectively in an oxidized state; however, its activity is inhibited when reduced by the selenoenzyme thioredoxin reductase (TrxR). (3) Latency and Reactivation: In latent infection, CD4^+^ T cells exhibit reduced glycolytic activity and diminished nicotinamide adenine dinucleotide phosphate (NADPH) production, lowering cellular reducing power and heightened sensitivity to oxidative stress. Inhibition of TrxR by compounds such as auranofin sustains Tat in an oxidized, transcriptionally active state, contributing to proviral reactivation. Abbreviations: ART, antiretroviral therapy; ETC, electron transport chain; HIV-1, human immunodeficiency virus type 1; NADPH, nicotinamide adenine dinucleotide phosphate; NF-κB, nuclear factor kappa-light-chain-enhancer of activated B cells; ROS, reactive oxygen species; Tat, transactivator of transcription; Trx, thioredoxin; TrxR, thioredoxin reductase. Image used with permission of ©Mount Sinai Health System Florence.

The Trx pathway is involved in multiple stages of the HIV-1 life cycle (Figure 1). During viral entry, thiol-disulfide exchange reactions are required for HIV-1 envelope glycoprotein (Env)–receptor interactions. Env (gp120/gp41) contains disulfide bonds that must isomerize for the conformational changes leading to fusion (Matthias and Hogg, 2003). Host cell-surface reductases, such as thioredoxin-1 and the protein disulfide isomerase (PDI), located on or near the cell membrane, can reduce critical disulfide bonds in gp120 and the CD4 receptor, thereby promoting viral entry (Matthias et al., 2002; Ou and Silver, 2006). For instance, Trx-1 was shown to selectively cleave the disulfide bond in the V3 loop of gp120, an action that enhances Env’s interaction with coreceptors (Azimi et al., 2010). Similarly, PDI and glutaredoxin-1 can reduce disulfides in gp120 or CD4, which is essential for membrane fusion and viral infection (Auwerx et al., 2009; Cerutti et al., 2014). Cell type–specific differences in thiol/disulfide exchange requirements have been observed, with Trx-1 preferentially involved in HIV-1 entry into macrophages and protein disulfide isomerase (PDI) preferentially involved in entry into resting CD4^+^ T cells (Stantchev et al., 2012). Inhibiting these thiol-reducing enzymes at the cell surface with small molecules or antibodies has been shown to block HIV-1 (Horváth et al., 2005), underscoring the importance of an oxidoreductase-rich environment for the initial stages of infection.

During the post-entry phase, thioredoxin relocates to the nucleus upon cellular activation, where it influences transcription (Figure 1). By keeping transcription factors in a reduced state, Trx promotes the DNA-binding activity of key proviral activators. At the same time, Trx (along with GSH) helps to buffer excess ROS generated by cell signaling or viral proteins, thereby protecting infected cells from oxidative stress-induced apoptosis. Indeed, moderate oxidative stress is known to activate specific pathways (e.g., NF-κB) that favor HIV-1 expression, but excessive ROS can trigger cell death; the Trx and GSH systems help navigate this fine line. The pivotal role of Trx and GSH in sustaining a conducive environment for HIV is highlighted by studies showing that dual inhibition of the Trx and GSH pathways has detrimental effects on the virus. *In vitro* and *in vivo*, the simultaneous suppression of Trx and GSH leads to elevated oxidative stress, which can selectively kill infected cells or render them more susceptible to immune clearance (Benhar et al., 2016). Conversely, supplementation of antioxidants alone does not cure the infection and can sometimes even favor the survival of infected cells. Taken together, these data suggest that HIV-1 thrives in a milieu of controlled redox imbalance–one in which pro-oxidant signals exist to drive viral gene activation but are counteracted sufficiently by host antioxidants to prevent cytotoxicity. The following sections will examine how this balance affects HIV-1 transcription and latency, as well as how the thioredoxin pathway interacts with cellular metabolism to regulate these processes.

## Redox control of HIV-1 transcription

Once HIV-1 has integrated into the host cell genome, the fate trajectory of latency or active replication is governed by host transcription factors and signaling pathways. The HIV-1 long terminal repeat (LTR) serves as a promoter region, containing binding sites for transcription factors such as NF-κB, AP-1, and NFAT, among others. Among these, NF-κB is a master regulator of HIV-1 transcription. Pro-inflammatory stimuli, such as TNF-α signaling, activate NF-κB by inducing the degradation of its inhibitor, IκB, allowing the NF-κB (p50/p65) heterodimer to translocate into the nucleus. Yet, the ability for NF-κB to bind DNA is redox-sensitive. The p50 subunit of NF-κB contains a critical cysteine (Cys62) in its DNA-binding domain that must be in the reduced (–SH) form for NF-κB to recognize κB sequences in the LTR. Trx-1 was identified as a key cofactor that enhances NF-κB DNA binding by reducing this cysteine residue (Matthews et al., 1992). *In vitro*, oxidizing Cys62 of p50 inhibits NF-κB binding to DNA, whereas the addition of Trx restores binding activity (Hayashi et al., 1993). Yodoi and colleagues demonstrated that Trx can activate the HIV-1 enhancer by thiol-reduction mechanisms (Okamoto et al., 1992). Thus, Trx translocation to the nucleus upon cell stimulation provides a redox control mechanism for HIV-1 transcription. Redox regulation is not limited to NF-κB. The AP-1 transcription factor family (c-Fos/c-Jun heterodimers) also contains cysteine residues in their DNA-binding domains and synergizes with NF-kB to promote proviral transcription via the HIV LTR. A cellular redox factor (Ref-1/APE1) and Trx together maintain AP-1 DNA binding ability by reducing critical cysteines (Schenk et al., 1994). In one study, Schenk and colleagues demonstrated that Trx specifically augmented NF-κB activity without similarly boosting AP-1, suggesting a role for Trx in HIV transcription that is tied explicitly to NF-κB redox control.

In addition to host transcription factors, viral proteins may also interface with redox pathways to modulate transcription. Tat augments RNA polymerase II processivity at the LTR. Intriguingly, Tat contains cysteine-rich regions, and the intracellular redox state influences its function. A highly oxidizing milieu or pharmacologic inhibition of TrxR can maintain Tat in an oxidized state, tuning its ability to bind the TAR RNA stem-loop or recruit transcriptional complexes (Figure 1). Tat also perturbs cellular redox homeostasis by generating oxidative stress in host cells. For instance, transgenic mice expressing HIV Tat in the lungs exhibited increased oxidant production and oxidative damage in pulmonary tissues (Cota-Gomez et al., 2011). In cultured cells, Tat can stimulate the production of reactive oxygen species (ROS) and reactive nitrogen species (RNS) by inducing NADPH oxidases and iNOS, and by depleting antioxidant defenses (Ali et al., 2021). Trx and TrxR help to counterbalance ROS levels associated with Tat expression. This delicate balance is exemplified in macrophages, where thioredoxin reductase-1 (TrxR1) has been found to negatively regulate HIV-1 transcription. In primary human macrophages, siRNA knockdown or pharmacological inhibition of TrxR1 resulted in enhanced Tat-driven LTR activity and increased HIV-1 expression, whereas high TrxR1 activity suppressed HIV transcription (Kalantari et al., 2008). Perhaps, lower TrxR1 activity in these cells allows for a transient increase in ROS, which boosts NF-κB/Tat activity, resulting in increased transcription. This concept aligns with the observation that auranofin, a gold-based inhibitor of TrxR, can provoke HIV reactivation in myeloid cells by tipping the redox balance. Beyond NF-κB and Tat, and further upstream in the antioxidant response is the master transcription factor Nrf2. Zhang and colleagues demonstrate that Nrf2 is upregulated in response to Tat and may act as a negative regulator of Tat-driven transcription at the HIV-1 LTR (Zhang et al., 2009). Moreover, some studies suggest a putative HIV-encoded peptide can bind free Trx by structural mimicry of NF-κB.

In summary, transcription of HIV-1 is highly responsive to cellular redox conditions. Therapeutically, this means that either antioxidative or pro-oxidative interventions can impact HIV-1 expression. General antioxidants, such as NAC and selenium compounds, tend to dampen NF-κB-driven HIV transcription (Roederer et al., 1992; Hori et al., 1997; Guillin et al., 2022), whereas mild pro-oxidants or inhibitors of cellular antioxidants can enhance HIV transcription (Gavegnano et al., 2019).

## Oxidative metabolism in HIV-1 infection and its redox interplay

HIV-1 infection disrupts mitochondrial oxidative phosphorylation (OXPHOS) and skews cells toward glycolysis and fatty acid synthesis, facilitating replication (Rodriguez et al., 2024). By contrast, antiretroviral therapy (ART) and chronic infection can lead to metabolic dysregulation, characterized by mitochondrial DNA depletion and increased reactive oxygen species (ROS) production as side effects (Rodriguez et al., 2024). Persistently infected individuals on ART often exhibit systemic inflammation and oxidative stress, linked in part to these metabolic perturbations. Distinct metabolic programs are also observed in cells that harbor latent HIV-1 versus active replication (Valle-Casuso et al., 2019). Resting memory CD4^+^ T cells rely predominantly on oxidative metabolism (fatty acid oxidation and OXPHOS) to meet their energy needs, in contrast to the glycolysis-fueled effector T cells. Recent single-cell transcriptomic studies in human tonsil models have demonstrated upregulation of oxidative phosphorylation programs in HIV-1-infected CD4^+^ T cells (Freeman et al., 2023; Schrode et al., 2024). Shytaj et al. (2021) demonstrated that the transition of an infected T cell into latency is marked by a downregulation of glycolysis, accompanied by lower glucose uptake and ATP production (Shytaj et al., 2021). Latently infected T cells exhibited a metabolic profile similar to that of quiescent cells and were significantly more susceptible to oxidative stress. Intriguingly, when these latent cells were exposed to TCR activation, their glycolytic activity was restored along with viral gene expression (Shytaj et al., 2021). These findings suggest that metabolic reprogramming is intimately tied to the maintenance of latency.

Yet, macrophages and monocytes exhibit distinct states of metabolic and redox balance. Upon monocyte differentiation into macrophages, there is typically an increase in metabolic activity, including OXPHOS and, depending on polarization, glycolysis (O'Neill and Pearce, 2016). In the context of HIV, monocyte-to-macrophage differentiation can influence latency. A recent study by Blanco et al. demonstrated that when latent HIV-infected monocytes differentiate into macrophages, they often undergo viral reactivation concomitant with the metabolic changes associated with differentiation (Blanco et al., 2024). This process was linked to the activation of protein kinase C signaling and the upregulation of Cyclin T1 (a cofactor for Tat), as well as shifts in redox homeostasis. Notably, polarization of macrophages had opposite effects on latency. Proinflammatory M1 macrophages (high ROS) suppressed HIV reactivation, whereas anti-inflammatory M2 macrophages (high NADPH/GSH) promoted reactivation.

The interplay between metabolism and redox in HIV-infected cells is complex. For example, high rates of glycolysis can support antioxidant capacity through the pentose phosphate pathway, generating NADPH needed for GSH and Trx regeneration. In HIV-infected T cells, metabolic and redox changes occur in tandem during the transition to latency or reactivation (Valle-Casuso et al., 2019; Shytaj et al., 2021). Likewise, in HIV-infected myeloid cells, metabolic reprogramming during differentiation affects the redox state, which in turn can trigger or suppress latent virus (Blanco et al., 2024).

## Integration of metabolic and redox signals: role of TXNIP

Thioredoxin-interacting protein (TXNIP) is an endogenous inhibitor of thioredoxin that binds to the reduced form of Trx and blocks its activity, thereby increasing intracellular ROS levels. Moreover, TXNIP is a key regulatory protein that links glucose metabolism to the cellular redox state (Kim et al., 2024; Parikh et al., 2007). In a recent study, Kim et al. (2024) demonstrated that when intracellular ROS levels exceed a threshold, TXNIP preferentially binds to glucose transporters (GLUT1) at the cell surface, triggering their internalization and subsequent lysosomal degradation. TXNIP effectively reduces glucose uptake by removing GLUT1 from the membrane, thereby suppressing glycolysis. Altogether, TXNIP switches its binding partner in response to redox changes, where high ROS prompts TXNIP to shift from Trx inhibition to GLUT inhibition. TXNIP has been identified as essential for the formation of long-lived memory Th1/Th2 cells by orchestrating a metabolic switch via the Nrf2–biliverdin reductase axis, which controls ROS levels (Kokubo et al., 2023). TXNIP may also impact HIV latency through interactions with the NLRP3 inflammasome under high glucose/ROS conditions, leading to IL-1β production (Zhou et al., 2010). In summary, TXNIP serves as a critical sensor and effector of metabolic-redox integration in cells, yet few studies have detailed its role in HIV-1 pathogenesis. Its modulation in HIV-infected cells represents a potential leverage point for therapies aiming to disturb the equilibrium of latency.

## Redox regulation of HIV-1 latency and reservoirs

Latently infected T cells have reduced antioxidant reserves and are sensitive to ROS-mediated apoptosis (Shytaj et al., 2021). Lundberg and colleagues demonstrated that PX-12 (1-methylpropyl 2-imidazolyl disulfide) significantly impedes HIV-1 infection in TZM-bl cells by inhibiting both Trx enzymatic activity and gp120 disulfide reduction (Lundberg et al., 2019). In latently infected primary T cells, PX-12 acts as a latency-promoting agent (LPA) (Blanco et al., 2024; Lu et al., 2021). Blanco et al. demonstrated a strikingly opposite effect of Trx system inhibition in macrophages versus T cells (Blanco et al., 2024). In their study, treatment with thioredoxin inhibitors PX-12 and auranofin produced divergent outcomes depending on the reservoir cell type. As shown in Figure 1, TrxR inhibition in CD4^+^ T cells and macrophages leads to opposing effects on HIV-1 reactivation, reflecting cell type–specific redox environments and Tat regulation. In macrophages, Trx system inhibition increased ROS to levels sufficient to stimulate NF-κB and other transcriptional activators, resulting in latency reversal and viral reactivation (Blanco et al., 2024). Auranofin, which, irreversibly inhibits TrxR, induced HIV expression in differentiated macrophages but not in T cells under the same conditions. These findings highlight the need for reservoir-targeted eradication strategies: CD4^+^ T cells and macrophages differ markedly in their baseline ROS levels, antioxidant capacity, and redox response to pharmacologic modulation.

Clinical trials administering antioxidants, such as NAC or selenium, to PLWH have shown minor improvements in T cell survival and function (Taylor et al., 2000; Breitkreutz et al., 2000). Auranofin in combination with intensified ART was tested in a pilot clinical trial of PLWH, and the drug was well tolerated and led to decreases in viral DNA in peripheral blood as well as proviral DNA in some individuals, consistent with an effect on the HIV reservoir (Diaz et al., 2019). Importantly, caution is warranted, as the therapeutic window between virus reactivation and toxic side effects is narrow when targeting redox homeostasis (Gavegnano et al., 2019).

## Future directions

Building on the mechanistic and translational insights reviewed above, the next phase of research on redox regulation in HIV-1 will require moving beyond descriptive studies toward mechanistic, cell type–resolved, and clinically translatable approaches. First, single-cell redox and metabolic profiling, leveraging high-parameter flow cytometry, metabolomics, and transcriptomics, should be applied to both blood and tissue reservoirs to define how redox states differ between CD4^+^ T cell subsets, myeloid-lineage cells, and anatomical compartments. Incorporating spatial transcriptomics and imaging-based ROS biosensors into *ex vivo* tissue models from PLWH could provide real-time maps of oxidative stress, antioxidant activity, and viral transcriptional states within intact microenvironments.

Second, there is a pressing need for longitudinal studies in PLWH on suppressive ART that track redox parameters in parallel with reservoir size, immune activation, and clinical outcomes. Such datasets could clarify whether shifts in redox balance precede, accompany, or result from viral reactivation events, and whether they are modifiable by diet, microbiome composition, or co-infections.

Third, translational work should focus on therapeutic precision in redox modulation. Preclinical findings already demonstrate that the same intervention, such as TrxR inhibition, can have opposite effects in T cells versus macrophages. Furthermore, evidence of cell-type specificity in thiol/disulfide exchange and HIV-1 entry, where Trx-1 is preferentially involved in virus entry of macrophages while PDI is preferentially involved in virus entry of resting CD4^+^ T cells, presents opportunities for tailoring therapies for effect against different tropism (CXCR4 vs. CCR5) and phase of infection (acute vs. chronic) (Stantchev et al., 2012). Future drug development should address this cell type specificity through targeted delivery systems, conditional activation of pro-drugs, or combination therapies that bias redox effects toward desired outcomes. Parallel safety assessments will be critical, as excessive ROS induction risks off-target tissue injury.

Finally, integrative, systems-level frameworks that combine redox biology, immunometabolism, and latency modeling are likely to yield the most impactful advances. This includes coupling latency reversal agents with metabolic modulators, exploiting redox-sensitive transcription factors as therapeutic nodes, and incorporating computational modeling to predict optimal intervention windows. By embracing these multidisciplinary strategies, the field can move closer to interventions that manipulate redox balance in a targeted manner, advancing efforts toward a functional cure or eradication of HIV-1.

## Conclusion

The thioredoxin pathway is involved in influencing viral entry, promoting or restraining transcription, and tipping the balance of HIV-1 latency. As summarized in Figure 1, the Trx pathway intersects with transcriptional regulation, redox buffering, and metabolic control at multiple points in the HIV-1 life cycle, making it a critical node in both viral pathogenesis and therapeutic targeting. HIV-1 has evolved to exploit a favorable redox window, and it co-opts host antioxidant systems, such as Trx and GSH. The thioredoxin pathway, by controlling the redox state of transcriptional regulators and buffering ROS, emerges as a master modulator of whether HIV genes remain off or switch on. Oxidative metabolism intersects with this pathway, as metabolic quiescence or activity sets the stage for how much oxidative stress a cell can handle and how it responds. Therapies aiming for an HIV cure must contend with these factors. They either stabilize the latent state via metabolic-redox reinforcement or dismantle it by transiently perturbing the redox environment. Importantly, what works for one reservoir cell type may not work for another, highlighting the need for tailored redox-modulating interventions. Future research is needed to unravel these complex interactions, such as dissecting the role of TXNIP and other redox sensors in HIV latency. Further integrating insights from redox biology and immunometabolism brings the field closer to interventions that can achieve functional cure or eradication of HIV-1.
